# Systematic Affiliation and Genome Analysis of *Subtercola vilae* DB165^T^ with Particular Emphasis on Cold Adaptation of an Isolate from a High-Altitude Cold Volcano Lake

**DOI:** 10.3390/microorganisms7040107

**Published:** 2019-04-23

**Authors:** Alvaro S. Villalobos, Jutta Wiese, Johannes F. Imhoff, Cristina Dorador, Alexander Keller, Ute Hentschel

**Affiliations:** 1 Marine Microbiology, GEOMAR Helmholtz Centre for Ocean Research Kiel, Düsternbrooker Weg 20, 24105 Kiel, Germany; avillalobos@geomar.de (A.S.V.); jwiese@geomar.de (J.W.); uhentschel@geomar.de (U.H.); 2 Laboratorio de Complejidad Microbiana y Ecología Funcional and Departamento de Biotecnología, Facultad de Ciencias del Mar y Recursos Biológicos, Universidad de Antofagasta, Av. Angamos 601, Antofagasta, Chile; cristina.dorador@uantof.cl; 3 Department of Bioinformatics, Biocenter, University of Würzburg, Am Hubland, 97074 Würzburg, Germany; a.keller@biozentrum.uni-wuerzburg.de

**Keywords:** cold adaptation, *Subtercola vilae*, genome analysis, systematic affiliation, Llullaillaco Volcano

## Abstract

Among the *Microbacteriaceae* the species of *Subtercola* and *Agreia* form closely associated clusters. Phylogenetic analysis demonstrated three major phylogenetic branches of these species. One of these branches contains the two psychrophilic species *Subtercola frigoramans* and *Subtercola vilae*, together with a larger number of isolates from various cold environments. Genomic evidence supports the separation of *Agreia* and *Subtercola* species. In order to gain insight into the ability of *S. vilae* to adapt to life in this extreme environment, we analyzed the genome with a particular focus on properties related to possible adaptation to a cold environment. General properties of the genome are presented, including carbon and energy metabolism, as well as secondary metabolite production. The repertoire of genes in the genome of *S. vilae* DB165^T^ linked to adaptations to the harsh conditions found in Llullaillaco Volcano Lake includes several mechanisms to transcribe proteins under low temperatures, such as a high number of tRNAs and cold shock proteins. In addition, *S. vilae* DB165^T^ is capable of producing a number of proteins to cope with oxidative stress, which is of particular relevance at low temperature environments, in which reactive oxygen species are more abundant. Most important, it obtains capacities to produce cryo-protectants, and to combat against ice crystal formation, it produces ice-binding proteins. Two new ice-binding proteins were identified which are unique to *S. vilae* DB165^T^. These results indicate that *S. vilae* has the capacity to employ different mechanisms to live under the extreme and cold conditions prevalent in Llullaillaco Volcano Lake.

## 1. Introduction

The genus *Subtercola* belongs to the family *Microbacteriaceae*, members of which are widely distributed in terrestrial and aquatic environments, or associated with macroorganisms [1]. Species of *Subtercola* together with those of *Agreia* form phylogenetically-related clusters. *Subtercola boreus* and *Subtercola frigoramans* are two psychrophilic species isolated from boreal groundwater in Finland [2]. 

Also *Subtercola vilae* was isolated from a cold water habitat, i.e., that of the Llullaillaco Volcano Lake [3]. *Subtercola lobariae* was isolated from the lichen *Lobaria retigera* in China [4]. While *Agreia* species appear specifically associated to plants and plant surfaces, species of *Subtercola* have been found in cold environments, including plant surfaces in such environments [5,6]. Species of both genera share a number of properties, and the borderlines between the two genera are not yet clearly defined. It should be mentioned that *Agreia pratensis* was originally classified as *Subercola pratensis* [7,8]. In view of these data, we have studied the genetic relationship between *Subtercola* and *Agreia* species in more detail, and analyzed the genome of *S. vilae* with a focus on aspects related to the cold-adaptation of this bacterium.

*S. vilae* DB165^T^ was isolated from Llullaillaco Volcano Lake at 6170 m above sea level (Llullaillaco Volcano, Chile) [3]. The environment has been characterized as a cold oligotrophic environment covered year-round by an ice layer. During different expeditions in summer time the lake was covered by an ice layer, while the temperature of lower water masses was at 3–4 °C (6.8 m depth). The temperature at the soil surface on a summer day may vary from −10 °C to >50 °C, due to the high solar radiation present [9,10]. A recent study demonstrated that the microbial communities of Llullaillaco soils are dominated by Actinobacteria, with more than 90% represented by members of the genus *Pseudonocardia* [10]. Bacteria affiliated to the class Actinobacteria are not only abundant in Llullaillaco soils, but also along the Andean mountains [10,11].

*S. vilae* revealed optimal growth at temperatures of 10 °C to 15 °C, but could grow well also at 5 °C. There was no growth at 30 °C [3]. These are characteristics of a psychrophilic bacterium. Taking into consideration the environmental distribution of the genus *Subtercola* in cold environments and the psychrophilic properties of *S. frigoramans* and *S. vilae*, the genome of *S. vilae* DB165^T^ was sequenced and genomic properties are reported, in particular concerning aspects with possible relevance to cold adaptation.

## 2. Materials and Methods

*S. vilae* DB165^T^ (DSM 105013^T^ = JCM 32044^T^) cells were grown in an SGG medium containing 10 g starch, 10 g glucose x H_2_O, 10 ml glycerol (99.7% v/v), 5 g soy peptone, 2.5 g corn steep solids, 2 g yeast extract, 3 g CaCO_3_, 1 g NaCl, and 18 g agar in 1 L of deionized water [12] for 5 days at 23 °C. DNA was extracted using a DNeasy®Blood&Tissue Kit (Qiagen, Hilden, Germany). The quality and quantity of the extracted DNA was evaluated by 0.8 % (w/v) agarose gel electrophoresis. The genomic DNA library was generated using Nextera XT (Illumina Inc., San Diego, CA, USA) according to the manufacturer’s instructions. After fragmentation, size-selection was performed using NucleoMag NGS Clean-up and Size Select (Macherey-Nagel, Düren, Germany) to obtain a library with median insert-size around 400 bp. After PCR enrichment, the library was validated with a high-sensitivity DNA chip and Bioanalyzer 2100 (both Agilent Technologies, Inc. Santa Clara, CA, USA), and additionally quantified using the Qubit dsDNA HS assay (Life Technologies, Ismaning, Germany). Four sequencing runs were performed on a NextSeq device (Illumina Inc., San Diego, CA, USA) using v2 2 × 150 bp chemistry. In total, 1,304,036,262 bp raw paired-end sequences were subjected to the Trimmomatic software for adapter and quality trimming (mean Phred quality score ≥ 30) [13] filtering of sequences containing ambiguous bases and a minimum length of 200 bp. The remaining 1,206,508,976 bp were assembled with SPAdes assembler, using enabled error pre-correction and k-mer sizes ranging from 15 to 127 (step size of 10) [14]. The assemblies obtained were analyzed using QUAST [15], whereas 127-kmers showed the best quality.

Open reading frames were identified using Prodigal in Prokka and barrnap for rRNA genes [16]. An additional gene prediction and functional annotation was performed with the Rapid Annotation using Subsystem Technology (RAST) webservers [17,18], and for natural product gene cluster a comprehensive resource for the genome mining of biosynthetic gene clusters, antiSMASH 3.0 was used [19]. The genome completeness was analyzed with CheckM [20]. A neighbor-joining phylogenetic tree was calculated using 16S rRNA gene of next related type strains of the *Microbacteriaceae* family, and sequences of strains annotated as *Subtercola* obtained from different environments. 

The sequences were aligned using SINA [21] and the tree was generated on MEGA6 [22] with 1000 bootstrap replicates. A maximum likelihood phylogenetic tree was calculated using 107 essential single-copy genes on bcgTree [23] with 1000 bootstrap. 

Putative ice-binding motifs in *S. vilae* DB165^T^ open reading frames were identified using blastp against amino acid sequences of anti-freeze proteins obtained from UniProt [24]. The hits were further analyzed by protein homology modeling using Phyre2 server [25] and corroborated according the position of functional threonine residues on the surface of the proteins using Visual Molecular Dynamic (VMD 1.9.3) software [26].

The sequencing project was completed in January 2017, and sequence data were deposited as a Whole Genome Shotgun (WGS) project in Genbank under the Bioproject PRJNA491396 and the accession number QYRT00000000, consisting of 103 contigs ≥1000 bp and N50 value of 87665. The version described in this paper is QYRT00000000. The annotated genome is available in RAST under the ID number 2056433.4. 

## 3. Results

### 3.1. Diversity and Relations of Subtercola and Related Agreia Species

**Figure 1:**
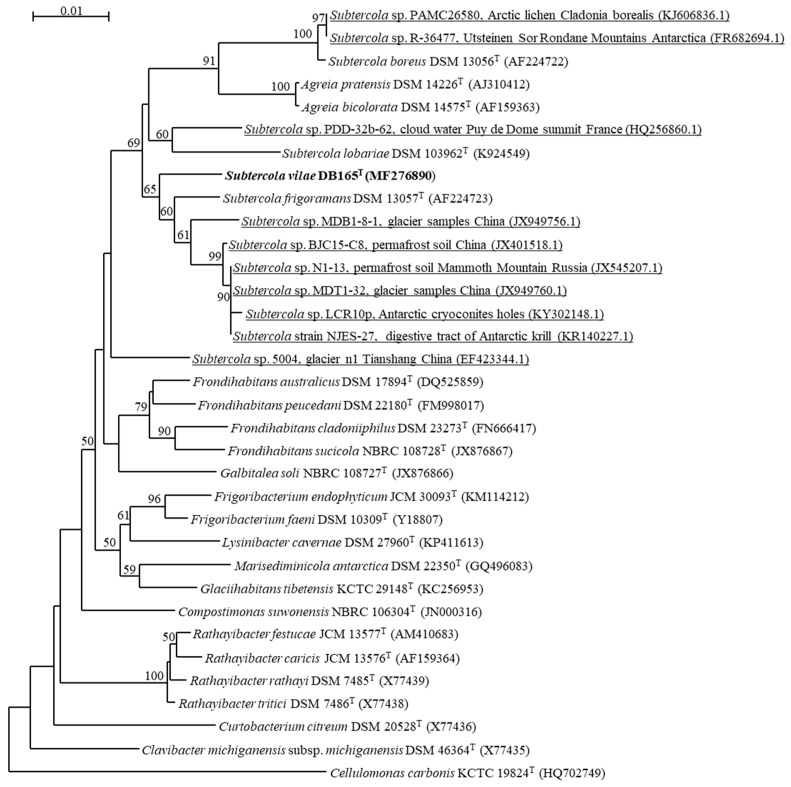
Phylogenetic tree based on the 16S rRNA gene sequences highlighting the position of *Subtercola vilae* DB165^T^ and other sequences annotated as *Subtercola* strains deposited in National Center for Biotechnology Information (NCBI), relative to phylogenetically closely-related type strains within the family *Microbacteriaceae* with 1000 bootstraps. Bootstrap values >50% are indicated. Bar indicates 0.05 nucleotides substitutions per site.

*Subtercola* and *Agreia* species constitute a clade well-separated from other Microbactericeae genera. The analysis of 16S rRNA gene sequences of *Agreia* and *Subtercola* species, including a number of new unclassified environmental isolates reveals interesting aspects on the environmental preferences (Figure 1). One major branch includes the group of *Agreia* species together with two distinct groups around *S. boreus* and *S. lobariae*. The association of many of the representatives of this branch to plants and lichens is remarkable. This includes all *Agreia* species and a number of additional environmental isolates, but also a *Subtercola* isolate from an Arctic lichen related to *S. boreus* as well as *S. lobariae* (Figure 1). In contrast, the second major branch includes *S. vilae* and *S. frigoramans* and a number of isolates from cold environments such as glaciers, Antarctic cryoconite holes, permafrost soils, Antarctic krill [6] (Figure 1), suggesting that these bacteria are adapted to their cold environments.

**Figure 2:**
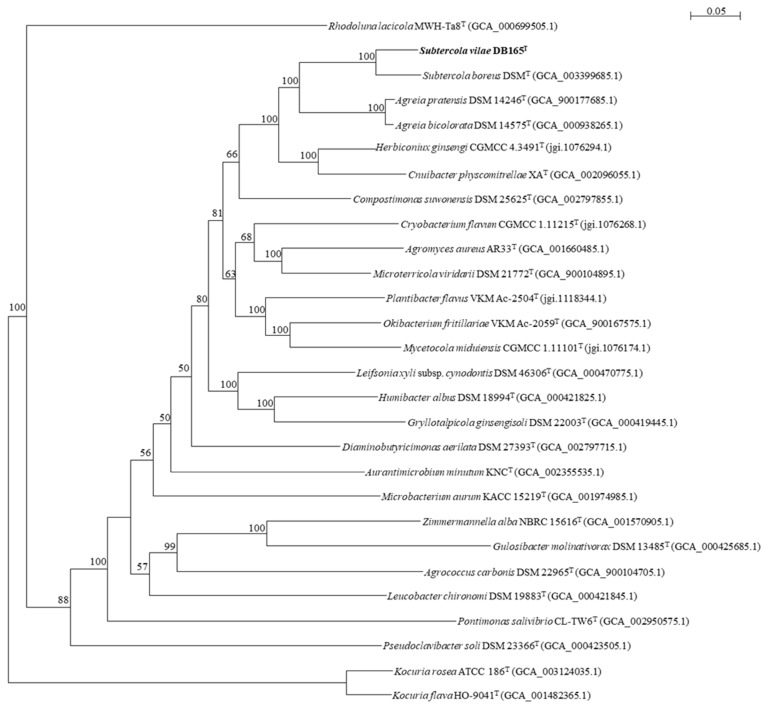
Maximum-likelihood tree based on 107 essential single-copy genes (amino acids) of *Subtercola vilae* DB165^T^ and *Microbacteriaceae*-type strains, and two *Kocuria*-type strains as outgroup with 1000 bootstraps. Project accession is indicated in brackets. Bootstrap values >50% are indicated. Bar indicates 0.05 amino acids substitutions per site.

Taken into consideration the available genome sequences, we have compared genomes of *Subtercola* and *Agreia* species using a selection of sequences from 107 single copy genes of various type strains of *Microbacteriaceae* (Figure 2). The maximum-likelihood phylogenetic tree of these essential single-copy genes demonstrates the clear differentiation between the *Subtercola* and *Agreia* species (*S. vilae* DB165^T^, *S. boreus* DSM 13056^T^, *Agreia bicolorata* DSM 14575^T^, and *A. pratensis* DSM 14246^T^). This separation is supported by high bootstrap values in each separation node, and with separation distances comparable to type species of other genera (e.g. *Herbiconiux*/*Cnuibacter*) (Figure 2). 

In support of this is the result from comparing the average nucleotide identity (ANI) of *S. vilae* DB165^T^ with *S. boreus* DSM 13056^T^ (78%) and with *A. bicolorata* DSM 14575^T^ (75%) and *A. pratensis* DSM 14246^T^ (75%), whereas the two *Agreia* species shared 87%.

### 3.2. Genome Properties

#### 3.2.1. General Properties

**Figure 3:**
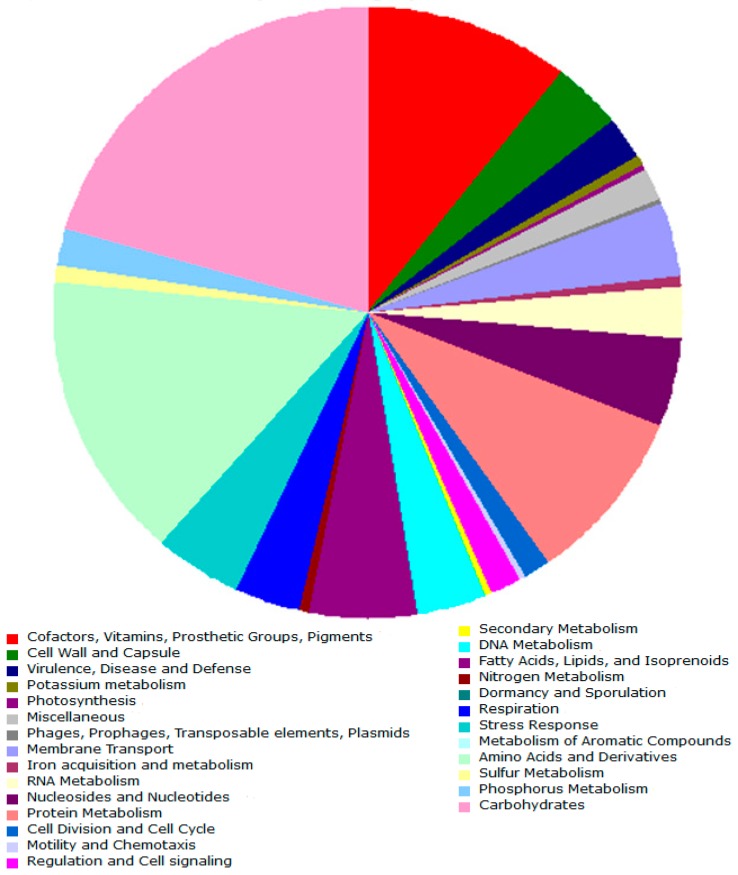
Metabolic subsystems of *S. vilae* DB165^T^ annotated through the RAST webserver.

The draft genome sequence of *S. vilae* DB165^T^ was assembled into 103 contigs (≥1000 bp) containing a total of 4,043,135 bp with an average G+C content of 65.1% (Table 1). From a total of 3879 predicted genes, 3797 (97.8 %) codify for proteins, and 2434 (62.7%) were annotated with a putative function. Genes not linked to a function were annotated as a hypothetical or unknown function. We annotated a total of 82 rRNA genes (2.1 %) divided into 5 rRNA genes (three 5S rRNA, one 16S rRNA, and one 23S rRNA) and 59 tRNA genes. Furthermore, a total of 1416 (36.5%) of the coding sequences were assignment to 24 different classes using COGs. Annotation obtained via RAST assigned a total 2089 sequences to 27 subsystem categories. The highest ranking among the subsystem categories are those concerned with the metabolism of carbohydrates (20.1 %), amino acids and derivatives (15.4 %), cofactors, vitamins, prosthetic groups, pigments (10.7 %), proteins (9.5 %), as well as fatty acids, lipids, and isoprenoids (5.1 %) (Figure 3). Completeness of the genome was calculated by CheckM, using the lineage marker set for Actinomycetales (UID1593), from which 99.5% of the proteins were present in the *S. vilae* DB165^T^ draft genome.

#### 3.2.2. Carbon and Energy Metabolism

According to the genomic repertoire of sugar metabolizing enzymes, *S. vilae* DB165^T^ has the potential to metabolize a wide range of sugars, ranging from monosaccharides such as mannose, D-ribose, xylose, D-gluconate ketogluconates, L-arabinose, D-galacturonate, and D-glucoronate; di- and oligosaccharides such as trehalose, sucrose, fructooligosaccharides (FOS), raffinose, maltose, maltodextrin, lactose, and galactose; the polysaccharides glycogen; sugar alcohols such as glycerol, glycerol-3-phosphate, mannitol, and inositol. *S. vilae* DB165^T^ has the potential to utilize the aminosugar chitin and its monomers, to convert them to fructose-6-phospate for the glycolysis pathway.

*S. vilae* DB165^T^ has encoded in its genome the enzymes 3-hydroxybutyryl-CoA dehydrogenase (Hbd), 3-hydroxybutyryl-CoA epimerase (Hbe), 3-ketoacyl-CoA thiolase (AtoB), and enoyl-CoA hydratase (Eh) needed for the production of poly-hydroxy-butyrates (PHB), that primarily serves as intracellular carbon and energy reserve [27], and are helpful against desiccation and osmotic stress, and also increase UV resistance [28]. The genome of *S. vilae* DB165^T^ also codifies for enzymes active in polyphosphate (PolyP) formation (exopolyphosphatase, polyphosphate kinase and polyphosphate glucokinase), serving in phosphate storage, but also in protection against UV radiation and temperature stress [29].

Three different rhodopsin genes are encoded in *S. vilae* DB165^T^. Two of them (fig|2056433.4.peg.750; fig|2056433.4.peg.1678) showed similarities in their amino acid sequences with annotated rhodopsin in *Subtercola boreus* (68%, WP_116415267.1; 66%, WP_116281575.1), distantly related to a bacteriorhodopsin of *Geodermatophilus* species (<50% similarity). The third one resembles a xanthorhodopsin [30] from *Clavibacter michiganensis* (81%, WP_079533889.1). The presence of rhodopsins in *S. vilae* DB165^T^ suggests that it might be able to transform energy from solar light [31].

#### 3.2.3. Secondary Metabolite Production

The antiSMASH analysis revealed the presence of three different secondary metabolite gene clusters for the biosynthesis of a type 3 polyketide, a terpene, and a cluster that contained core biosynthetic genes for the non-ribosomal peptide synthesis pathway, but was not categorized in this compound family.

The detected polyketide type 3 synthetase cluster has a total of 36 genes, where 3 genes were identified as the alkylresorcinol synthetic cluster, showing the same sinteny as found in *Agreia* species, while other annotated genes were affiliated to aminotransferase class V amidase, short chain dehydroganases/reductase, aldo-/ketoreductase family oxidoreductases, pullulanase type I, and alpha-glucosidase, suggesting modifications in the alkylresorcinol scaffold. The genes found in this cluster showed a 63% resemblance to a polyketide type-3 cluster found in *Agreia* sp. Leaf335, and 55% of genes showed similarity with *A. bicolorata* VKM Ac-1804T. This indicates the possible biosynthesis of alkylresorcinols by *S. vilae*. These compounds can easily be incorporated into the cell membranes, causing considerable changes in their structure and properties [32]. Some also showed antibiotic activity [33]. 

The terpene biosynthetic cluster consists of 24 genes identified as the carotenoid biosynthetic clusters. Genes annotated as core biosynthetic genes were determined as phytoene synthase, lycopene beta elongase BC, while additional biosynthetic genes include polyprenyl synthetase, dehydrogenase, and a short-chain dehydrogenase/reductase. The carotenoids in *S. vilae* DB165^T^ might play an important role in light protection, as membrane modulators at low temperatures [34] and also as antioxidants [35]. 

The third uncategorized gene cluster has a total of 13 genes and revealed a gene structure, which apparently is conserved in *Microbacteriaceae* species of the genera *Agreia*, *Clavibacter*, and *Cellulomonas*. The core biosynthetic gene encodes an amino acid adenylation protein similar to the one found in the saframycin A biosynthetic gene cluster, and a zinc metalloprotease. An additional biosynthetic gene, encoding 4-aminobutyrate aminotransferase, showed identity with the one present in the saframycin A biosynthetic gene cluster. 

### 3.3. Cold stress Adaptation of Subtercola Vilae DB165^T^

In order to identify adaptation to the harsh conditions of the Llullaillaco Volcano environment, annotated genes sorted in the subsystem categories of RAST and an in-house database of ice-binding motifs were used. Analysis of tRNA species, predicted using barrnap, showed that *Subtercola* strains isolated from cold waters, showed a higher number of tRNA genes if compared to others. While *S. vilae* DB165^T^ encodes 59 and *S. boreus* DSM 13056^T^ 53 tRNA genes, the *Agreia* species found in nematodes and plants revealed only 51 (*A. bicolorata* DSM 14575^T^) and 50 (*A. pratensis* DSM 14246^T^) such genes. The higher number and diversity of tRNAs might help counteracting their slow mobilization to the translation sites in those species of *Subtercola* adapted to cold waters [36,37].

#### 3.3.1. Cryoprotectants

Genes involved in production and uptake of choline and glycine betaine were found in the *S. vilae* DB165^T^ genome. These compounds are able to maintain membrane fluidity at low temperature and also prevent cold-induced aggregation of cellular proteins [38]. Intracellular proteins can also be protected by the production of the sugar trehalose [39], and *S. vilae* encodes the complete pathway for the biosynthesis of trehalose and utilization of trehalose. In addition, *S. vilae* DB165^T^ contains 11 copies of a trehalose permease transport system (SugB).

#### 3.3.2. Temperature Shifts

Among the functions possibly involved in the survival at low temperature are cold shock proteins. The fast production of such cold-inducible proteins is an important adaptation to low temperatures [40]. In *S. vilae* DB165^T^ we found cold-shock (Csp) proteins annotated as CspA and CspC on RAST and Prokka as well. These proteins act as RNA chaperones, which destabilize mRNA secondary structures formed at low temperatures, and enhance translation efficiency [41]. The genes encoding these proteins are shared between *S. vilae* DB165^T^ and *S. boreus* DSM 13056^T^. On the other hand, we found 11 heat shock proteins that prevent denaturation of cellular proteins at high and low temperatures [42].

#### 3.3.3. Oxidative Stress

Metabolic reactive oxygen species (ROS) generate intracellular damage in proteins, membranes, and DNA. More dissolved oxygen can be found in the water at low temperatures, and may increase the potential of possible damage [43]. Psychrophilic microorganisms are adapted to this harsh condition, and one of these adaptations against the damage caused by ROS is the production of different enzymes involved in the detoxification of the superoxide radical (O_2_−). *S. vilae* DB165^T^ encodes several enzymes to fight against the ROS stress. The enzymes found are deferrochelatase/peroxidase (EfeN), thioredoxin/glutathione peroxidase (BtuE), deferrochelatase/peroxidase (EfeB), putative heme-dependent peroxidase, catalase-peroxidase (KatG: one copy), putative non-heme bromoperoxidase (BpoC), catalases (KatA), and superoxide dismutases [Mn; Fe; Cu-Zn] (SodABC).

#### 3.3.4. Ice-binding Proteins

**Figure 4:**
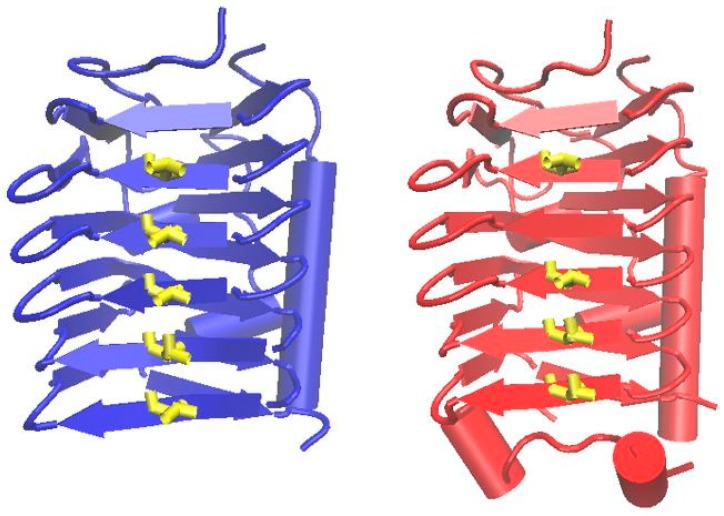
Cartoon representation of ice-binding motif models of Svilae_00062 (blue) and Svilae_00202 (red). The putative ice-binding surface with ordered threonine residues (yellow), is shown. Arrows and ribbons represent β-strands and α-helices, respectively.

In order to understand possible mechanisms to cope with ice crystal formation inside the cell, we screened the amino acid sequences of all *S. vilae* DB165^T^ genes against an in-house ice-binding motif database. We obtained two hits of hypothetical proteins with an ice-binding motif in the *S. vilae* DB165^T^ genome: Svil_00062, which consist in 389 amino acids and Svil_00202, with 380 amino acids. The comparison of both proteins against the Genbank database using blastp showed low identity (40–43%), and low query coverage, sharing only 50–52% of the amino acid sequence with proteins annotated as hypothetical, as well as DUF3494 domain-containing in genomes of other Actinobacteria, such as *Arthrobacter alpinus* (WP_074712914.1), *Cryobacterium* sp. Hh39 (WP_119975291.1), *Nocardia vaccinia* NBRC 15922^T^ (WP_084525865.1), *Streptomyces xinghaiensis* S187^T^ (WP_039820269.1), among others. The DUF3494 domain has been characterized as an ice-binding motif, suggesting that this conserved motif in Svil_00062 and Svil_00202 proteins may confer the function to bind with ice, whereas the other part of the proteins is not similar with anything reported in the database. As the ice-binding domain revealed a low identity with DUF3494 domain-containing proteins (40–45%) of the database, the sequences of both proteins were modeled by homology with proteins with known structure using the Phyre2 server. The result showed that the proteins were structurally similar to the antifreeze protein observed in the bacterium *Colwellia psychrerythraea* 34H, which was used as backbone with 100% of confidence for both proteins. The pattern in the β-strands and α-helices of the protein, and the threonine residues, are displayed in parallel, and are facing outside the β-strands (Figure 4). As the placement of these residues is considered to be essential for the ice binding function, it can be concluded that both proteins may have the predicted function [44].

As the other part of the proteins did not show hits with high coverage and identity, the two proteins quite likely represent a new type of ice-binding protein. Interestingly, we could not find either of the two proteins in the genomes of *S. boreus* DSM 13056^T^, *A. bicolorata* DSM 14575^T^, and *A. pratensis* DSM 14246^T^, which are the closest related type strains to *S. vilae* DB165^T^.

## 4. Conclusions

The comparison of 107 selected essential single-copy genes from *S. vilae* DB165^T^ and the type strains of the closely-related species *S. boreus*, *A. bicolorata*, and *A. pratensis*, revealed a clear separation of the *Subtercola* and *Agreia* species (Figure 2). This is in line with the separate branch of *Agreia* species seen with 16S rRNA gene sequences (Figure 1), and the ecological preference of *Agreia* for plant surfaces and of *Subtercola* species for cold environments. Among these species, in particular *S. vilae* appears to be adapted to live in a cold environment. Several properties of the genome of *S. vilae* DB165^T^, which was isolated from the high-altitude cold Llullaillaco Volcano Lake, support this view. This is the first specific study of a genome from such an extreme and cold environment, with particular focus on genomic properties possibly related to the cold adaptation.

**Figure 5:**
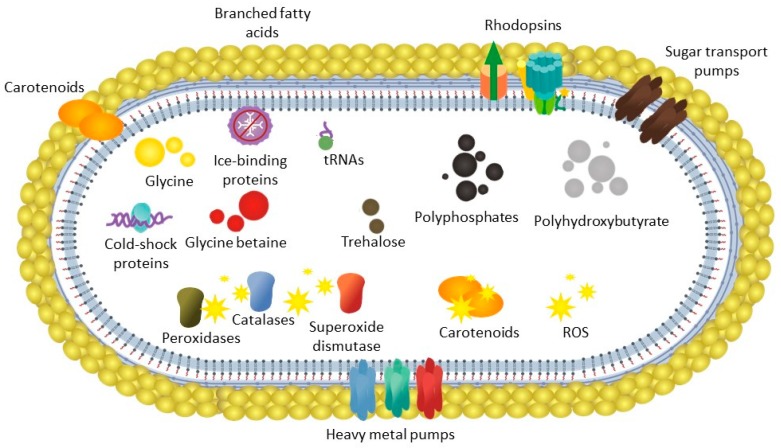
Traits annotated in the *S. vilae* DB165^T^ genome considered to be involved in its adaptation to the Llullaillaco Volcano Lake. ROS, reactive oxygen species.

The genome encodes a repertoire of genes. The features encoded in the genome of *S. vilae* DB165^T^, related to adaptation to the harsh condition of this Llullaillaco Volcano Lake, include mechanisms to transcribe proteins under low temperatures, such as a high number of tRNAs and cold shock proteins as well (Figure 5). *S. vilae* DB165^T^ is capable of producing several proteins to deal with oxidative stress, which is of higher relevance at low-temperature environments, in which reactive oxygen species are more abundant.

Of particular importance for adaptation to the low-temperature conditions is the ability to produce and take up metabolites that prevent cold-induced aggregation of cellular macromolecules, and also assist in maintaining membrane fluidity [38,45,46,47]. Such solutes, also known as compatible solutes, are represented by glycine betaine and also trehalose, both of which can be synthesized by *S. vilae*.

Most important for adaptation to the environment with regular ice crystal formation due to great daily changes in temperature appears the possibility of avoiding the formation of ice crystals within the cells, and the production of ice-binding proteins. To combat against the ice crystal formation, two new ice-binding proteins were identified with specific ice-binding domains similar to those found in *Colwellia psychrerythraea*. These ice-binding proteins are uniquely present in *S. vilae* DB165^T^, but absent from the related *Subtercola* and *Agreia* species *S. boreus* DSM 13056^T^, *A. bicolorata* DSM 14575^T^, and *A. pratensis* DSM 14246^T^. 

Altogether, the presented data demonstrate that *S. vilae* DB165^T^ can employ an array of different strategies to live at cold temperatures, as prevalent in Llullaillaco Volcano Lake, and can cope with the various stress factors prevailing in the cold environment. 

## Figures and Tables

**Table 1:** Genome statistics according to Prokka annotation.

Attribute	Value	Percentage of total
Genomes Size (bp)	4,043,135	100
Contigs	103	
N50	87,665	
DNA G+C content	65.1	
Total of genes	3879	100
Coding sequences	3797	97.8
Genes with function prediction	2434	62.7
Genes assigned to COGs	1416	36.5
RNA genes	82	2.11
rRNA genes	5	0.1
Pseudo genes	0	0
5S rRNA	3	0.07
16S rRNA	1	0.02
23S rRNA	1	0.02
tRNA	59	1.5
Other RNA	18	0.46
